# Young hearts, early risks: novel cardiovascular biomarkers in former very preterm infants at kindergarten age

**DOI:** 10.1038/s41390-024-03210-7

**Published:** 2024-04-24

**Authors:** Wolfgang Mitterer, Irena Odri Komazec, Eva Huber, Benedikt Schaefer, Anna Posod, Ursula Kiechl-Kohlendorfer

**Affiliations:** 1 Department of Pediatrics II, Medical University of Innsbruck, Innsbruck, Austria; 2 VASCage GmbH, Research Centre on Vascular Ageing and Stroke, Innsbruck, Austria; 3 Department of Pediatrics III, Medical University of Innsbruck, Innsbruck, Austria; 4 Department of Medicine I, Medical University of Innsbruck, Innsbruck, Austria

## Abstract

**Background:**

Preterm birth is associated with long-term cardiovascular morbidity and mortality. In adults, fibroblast growth factor-23 (FGF-23), α-Klotho, and secretoneurin have all garnered attention as cardiovascular biomarkers, but their utility in pediatric populations has not yet been ascertained. The aim of this pilot study was to evaluate these novel cardiovascular biomarkers and their association with indicators of cardiovascular impairment in the highly vulnerable population of former very preterm infants.

**Methods:**

Five- to seven-year-old children born at < 32 weeks’ gestation were eligible for the study. Healthy same-aged children born at term served as controls. Biomarkers were quantified in fasting blood samples, and echocardiographic measurements including assessment of aortic elastic properties were obtained.

**Results:**

We included 26 former very preterm infants and 21 term-born children in the study. At kindergarten age, former very preterm infants exhibited significantly higher plasma concentrations of biologically active intact FGF-23 (iFGF-23; mean 43.2 pg/mL vs. 29.1 pg/mL, *p* = 0.003) and secretoneurin (median 93.8 pmol/L vs. 70.5 pmol/L, *p* = 0.046). iFGF-23 inversely correlated with distensibility of the descending aorta.

**Conclusion:**

In preterm-born children, iFGF-23 and secretoneurin both offer prospects as valuable cardiovascular biomarkers, potentially allowing for risk stratification and timely implementation of preventive measures.

**Impact:**

- Former very preterm infants have increased plasma concentrations of the novel cardiovascular biomarkers intact fibroblast growth factor-23 (iFGF-23) and secretoneurin at kindergarten age.
- Increases in iFGF-23 concentrations are associated with decreased distensibility of the descending aorta even at this early age.
- Monitoring of cardiovascular risk factors is essential in individuals with a history of preterm birth. Both iFGF-23 and secretoneurin hold promise as clinically valuable biomarkers for risk stratification, enabling the implementation of early preventive measures.

## Introduction

According to the World Health Organization (WHO), cardiovascular disease is responsible for an estimated 17.9 million (32%) deaths in 2019 and is, therefore, the single leading cause of global mortality.^1^ While family history and lifestyle factors are undeniably linked to the development of cardiovascular disease, preterm birth (i.e., < 37 weeks’ gestation), affecting 10.6% of all livebirths worldwide,^2^ has been recognized as a significant determinant contributing to the formation of long-term cardiovascular morbidity^3^ and mortality.^4–7^ Preterm birth is associated with elevated blood pressure^8–12^ and hypertension,^8,13,14^ diabetes mellitus type 1 and 2,^4,15,16^ reductions in elastic properties of the aorta,^17^ ischemic heart disease,^18^ risk of heart failure,^19^ stroke,^20^ chronic kidney disease^21^ and cardiovascular mortality^4–6^ in early childhood and young adulthood. A link between childhood cardiovascular risk factors and adult cardiovascular events has just recently been demonstrated in a long-term prospective cohort study.^22^

In light of the mounting burden of prematurity and its association with cardiovascular disease, a necessity arises to find biomarkers capable of identifying those at increased risk to enable timely therapeutic intervention. In adult research, fibroblast growth factor-23 (FGF-23) and its co-receptor α-Klotho have attracted interest as promising biomarkers of cardiovascular disease.^23^ High concentrations of FGF-23 are associated with hypertension,^24^ left ventricular mass and hypertrophy,^25–27^ incident coronary heart disease, heart failure and overall cardiovascular mortality.^28–31^ Animal studies further suggest direct – i.e. Klotho-independent – cardiotoxicity of FGF-23^32,33^ and an association with reduced aortic relaxation.^34^ Low levels of soluble α-Klotho are associated with congestive heart failure and myocardial infarction.^35^ This increasing body of evidence leads to the question whether FGF-23 and α-Klotho may be suitable markers of an increased cardiovascular risk or even causal contributory factors in cardiovascular disease.^23^ Along similar lines, secretoneurin proved valuable in predicting mortality in patients with severe sepsis and septic shock,^36,37^ cardiovascular-related acute respiratory failure,^38^ acute and chronic heart failure^39,40^ and in patients undergoing heart surgery due to structural heart disease.^41,42^ Animal models further indicate an effect of secretoneurin on endothelium-dependent vascular relaxation.^43^ A link to the above-mentioned FGF-23 pathway may exist via respective ties to hypoxia-inducible factor 1-alpha (HIF-1α), as evidenced in both in vitro and in vivo models of hypoxia.^44,45^ Repeated hypoxic episodes during the vulnerable preterm period may alter HIF-1α circuits leading to downstream changes in FGF-23, α-Klotho, and secretoneurin, respectively.

The aim of the present study was to explore these novel cardiovascular biomarkers and their association with incipient end-organ impairment by means of non-invasive echocardiographic measurements in a population of very preterm infants.

## Methods

### Study design and population

The study was carried out at the Department of Pediatrics II (Neonatology), Innsbruck University Hospital, Austria. The following data was drawn from a group of former preterm infants born between 01/01/2007 and 07/31/2009 at < 32 weeks’ gestation (i.e., very preterm infants), who were invited to a routine visit at our preterm outpatient follow-up clinic at kindergarten age. A control group of same-aged children born at term were recruited through local kindergartens. None of the participants had congenital malformations or chromosomal abnormalities. In accordance with the Declaration of Helsinki in its most recent form and the International Conference on Harmonization: Good Clinical Practice (ICH-GCP) guidelines, approval for this study was obtained in advance from the institutional review board (IRB) of the Medical University of Innsbruck (UN 4491). Written informed consent was obtained from legal guardians, and all children verbally consented to participation.

### Perinatal characteristics

Basic perinatal data for preterm-born participants were drawn from our institution’s routine preterm follow-up database. To account for sex- and gestational age-specific differences, z-scores for birth weight were calculated by means of Fenton 2013 Growth Calculator for Preterm infants (available from http://www.peditools.org/fenton2013).^46^ Classification of smoking during pregnancy was based on self-reporting by mothers. Maternal educational status was classified as less than 12 years or 12 years and more. Perinatal data from children born at term previously not registered at our hospital and data on preterm-born children missing from our preterm follow-up database was filled in at study visit. The remaining missing data was classified as “unknown”.

### Study visit

All examinations were carried out by specifically trained staff at either Innsbruck University Hospital or provisional medical posts installed at participating kindergartens between 8 and 10 a.m. After a routine clinical examination, body weight was measured using calibrated medical scales and height was determined by a Harpenden stadiometer. Body mass index (BMI) was calculated as weight in kilograms divided by height in meters squared. To account for sex- and age-specific differences, z-scores for BMI were calculated by means of a reference data set.^47^ Blood pressure was measured three times on the right upper extremity after a five-minute resting period in a seated position using the appropriate cuff size and an automated oscillometric device. All children and their parents were asked to fill out a questionnaire on family history of cardiovascular disease and childhood nutrition habits. A positive family history of cardiovascular disease was defined as a diagnosis of coronary heart disease, angina, heart attack, congenital heart disease or stroke in first-degree male relative of child and/or parent under the age of 55 or first-degree female relative of child and/or parent under the age of 65. Information on childhood nutrition habits was collected by means of an established standardized food frequency questionnaire (“What do you eat?”, kindly provided by the Robert Koch Institute, Berlin, Germany).^48^ Nutrition habits were subsequently categorized as “unfavorable”, “neutral” or “favorable” for further analyses.

Blood sampling was performed after a minimum overnight fasting period of eight hours. Routine laboratory analyses were conducted at the Central Institute for Medical and Chemical Laboratory Diagnosis at Innsbruck University Hospital. The estimated glomerular filtration rate (eGFR) was calculated by means of the revised Schwartz Formula.^49^ Concentrations of HIF-1α in plasma were determined by using a solid phase sandwich enzyme-linked immunosorbent assay (ELISA) according to manufacturer’s instructions (HIF-1α ELISA Kit; EHIF1a, Invitrogen by Thermo Fisher Scientific, Vienna, Austria; inter-assay imprecision coefficient of variability (CV) < 10%). Biologically active intact FGF-23 (iFGF-23) was quantified by an ELISA that detects both N- and C-terminal fragments (Kainos Laboratories, Tokyo, Japan; inter-assay imprecision CV < 10%). The cleavage product C-terminal FGF-23 (cFGF-23) was measured using an ELISA capable of detecting epitopes within the carboxyl-terminus of FGF-23 with polyclonal antibodies (Biomedica, Biomedica Medizinprodukte, Vienna, Austria; inter-assay imprecision CV < 10%). Soluble α-Klotho concentrations in plasma were determined by means of an ELISA according to the manufacturer’s instructions (human soluble α-Klotho assay kit JP27998, Immuno-Biological Laboratories Co., Gunma, Japan; inter-assay imprecision CV < 11.4%). Secretoneurin concentrations were determined by using a sandwich ELISA according to the manufacturer’s instructions (CardiNor AS, Oslo, Norway; inter-assay imprecision CV < 10%). For all blood parameters, values below the respective lower limits of detection (LOD) were censored as LOD/√(2). Both term- and preterm-born participants underwent standard pediatric transthoracic echocardiography. As previously described,^17^ aortic elastic properties were calculated from transthoracic M-mode echocardiographic images using a standardized ultrasound protocol and software tool for computerized wall contour analysis.^50,51^

### Statistical methods

Statistical analyses were performed with IBM SPSS Statistics software, version 27.0.1 (IBM Corp., Armonk, NY). Categorical variables were expressed as absolute numbers (%) and analyzed by means of Fisher’s Exact or Chi-Square Test. Shapiro-Wilk and Kolmogorov-Smirnov Tests were used to assess the normal distribution of continuous data. Normally distributed variables were reported as mean ± standard deviation (SD) and analyzed for differences between groups using Student’s T-Test, whereas variables following a non-normal distribution were described as median with 1st and 3rd quartiles and analyzed using Mann-Whitney U Test. To ensure representativeness of study samples, both term and preterm study cohorts were compared to reference populations (term: SIDS database Tyrol, birth dates 01/01/2007 – 07/31/2009; preterm: Innsbruck routine preterm follow-up database, birth dates 01/01/2007–07/31/2009) regarding sex distribution, gestational age, and birth weight by means of Fisher’s Exact Test and Mann-Whitney U or Student’s T-Test, depending on type and distribution of the variable analyzed. Bivariate correlation and simple linear regression models were performed to determine possible relationships between biomarkers and echocardiographic measurements. Missing data for distinct variables are reported in the respective Table captions. A two-sided alpha level < 0.05 was considered significant for all statistical analyses.

## Results

### Perinatal characteristics and characteristics at study visit

A total number of 47 participants, 26 former very preterm infants (female 53.8%, mean gestational age 29.5 weeks) and 21 term-born children (female 47.6%, mean gestational age 40.3 weeks), were included. Except for gestational age at birth and birth weight, no differences in perinatal data and characteristics at study visit between preterm and term infants were found. Details are provided in Table 1. When comparing study participants to reference populations of preterm and term infants born within the same timeframe, no differences were found regarding distribution of sex, birth weight and gestational age for preterm infants and sex and birth weight for term infants, respectively (Supplementary Materials, Supplementary Tables 1 and 2).

**Table 1:** Perinatal characteristics and characteristics at study visit in former term and very preterm infants.

Characteristics	Term (*n* = 21)	Preterm (*n* = 26)	*p*-value
Sex, male/female, *N* (%)	11 (52.4) / 10 (47.6)	12 (46.2)/14 (53.8)	0.772
**Perinatal characteristics**			
Gestational age [weeks]	40.3 (38.7; 40.9)	29.5 (26; 31.4)	< 0.001***
Birth weight [g]	3345 ± 446	1204 ± 413	< 0.001***
Birth weight z-score	−0.29 ± 0.85	−0.03 ± 0.8	0.286
Maternal smoking during pregnancy, *N* (%)	2 (9.5)	4 (15.4)	0.079
Maternal educational status, unknown / < 12 years / ≥ 12 years, *N* (%)	4 (19.0) / 9 (42.9) / 8 (38.1)	0 (0.0) / 15 (57.7) / 11 (42.3)	0.064
**Characteristics at study visit**			
Age at examination [years]	5.6 (5.3; 6.0)	5.4 (5.3; 5.5)	0.131
Current BMI [kg/m²]	14.6 (14.1; 15.5)	14.3 (13.6; 15.0)	0.058
Current BMI z-score	−0.56 (−1.01; 0.08)	−0.70 (−1.39; −0.41)	0.080
Systolic blood pressure [mmHg]	97 ± 4	99 ± 5	0.275
Diastolic blood pressure [mmHg]	55 ± 7	55 ± 8	0.902
Positive family history of cardiovascular disease, *N* (%)	1 (4.8)	1 (3.8)	0.534
Favorable childhood nutrition, *N* (%)	15 (71.4)	12 (46.2)	0.137
**Routine laboratory parameters at study visit**			
Plasma urea [mg/dL]	25.7 ± 5.7	24.1 ± 6.8	0.418
Plasma creatinine [mg/dL]	0.43 ± 0.05	0.42 ± 0.05	0.538
eGFR (Schwartz) [ml/min/1.73 m^2^]	113 (102; 122)	111 (104; 126)	0.831
C-reactive protein [mg/dL]	0.07 (0.04; 0.12)	0.04 (0.04; 0.16)	0.599
Interleukin-6 [ng/L]	3.0 (2.3; 5.4)	3.5 (2.6; 6.3)	0.591
Red blood cell count [T/L]	4.75 ± 0.29	4.82 ± 0.32	0.436
Hemoglobin [g/L]	130 ± 8	131 ± 9	0.697
Hematocrit [L/L]	0.367 ± 0.02	0.369 ± 0.02	0.758
MCV [fL]	77.9 (75.4; 79.9)	77.0 (75.7; 78.7)	0.714
MCH [pg]	27.6 (26.9; 28.4)	27.6 (26.8; 28.1)	0.965
MCHC [g/L]	356 (347; 362)	356 (351; 362)	0.556
RDW [%]	13.0 (12.8; 13.3)	13.3 (12.7; 13.5)	0.356
Serum iron [µmol/L]	14.8 ± 6.4	16.8 ± 5.5	0.267
Serum transferrin [mg/dL]	272 ± 33	268 ± 31	0.727
Serum transferrin saturation [%]	22 ± 10	25 ± 9	0.291
Serum ferritin [µg/L]	43 (32; 55)	44 (30; 56)	0.877
Plasma total calcium [mmol/L]	2.46 ± 0.08	2.46 ± 0.08	0.943
Plasma phosphate, inorganic [mmol/L]	1.46 ± 0.17	1.37 ± 0.16	0.056
Alkaline phosphatase [U/L]	222 ± 50	229 ± 47	0.647

Categorical data are presented as counts (*N*) and percentages; continuous data are presented as median (quartile 1; quartile 3) for non-normally distributed variables or mean ± standard deviation (SD) for variables following a normal distribution. Data for C-reactive protein (limit of detection (LOD) 0.06 mg/dL) and interleukin-6 (LOD 2.0 ng/L) analyses were censored as follows: LOD/√(2). ***p *<* 0.001.

*BMI* body mass index (calculated as weight in kilograms divided by height in meters squared), *eGFR* estimated glomerular filtration rate (calculated by the revised Schwartz pediatric bedside formula (2009) as eGFR = 0.413 X (height/serum creatinine), where height is in cm, and creatinine is in mg/dL), *MCV* mean corpuscular volume, *MCH* mean corpuscular hemoglobin, *MCHC* mean corpuscular hemoglobin concentration, *RDW* red cell distribution width*.*

### Routine laboratory analyses at study visit

We did not detect significant differences in routine laboratory parameters between preterm- and term-born children at study visit. Both groups had normal kidney function, and showed no signs of inflammation, anemia/increased erythropoiesis or iron deficiency. Plasma phosphate concentrations were lower in the preterm group, but this finding did not reach statistical significance (*p* = 0.056). Plasma total calcium and alkaline phosphatase did not differ between groups. Details are shown in Table 1.

### HIF-1α and novel cardiovascular biomarkers at study visit

**Fig. 1:**
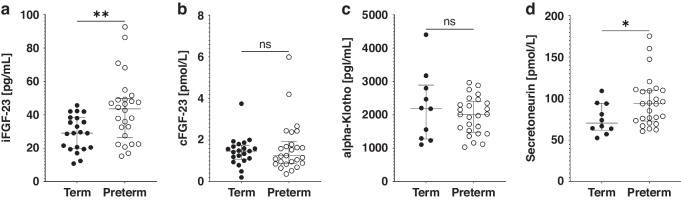
Novel cardiovascular biomarkers in term- and very preterm-born children at kindergarten age. Plasma concentrations of intact fibroblast growth factor-23 (iFGF-23) **a**, c-terminal FGF-23 (cFGF-23) **b**, α-Klotho **c**, and secretoneurin **d** are shown. Each circle represents an individual measurement. Measurements in term-born children are depicted as black circles, measurements in very preterm-born children as white circles. Biomarker concentrations are plotted on the y-axis in pg/mL and pmol/L, respectively. Center lines represent medians, whiskers mark 1st and 3rd quartiles. In comparison to term-born children, former very preterm infants had significantly higher iFGF-23 and secretoneurin plasma concentrations. **p < 0.01, *p < 0.05; ns not significant.

Plasma measurements were available in 47 of 47 subjects for HIF-1α, iFGF-23, and cFGF-23, 35 of 47 subjects for α-Klotho (preterm: 25/26 (96.2%), term: 10/21 (47.6%)), and 36 of 47 subjects for secretoneurin (preterm: 26/26 (100%), term: 10/21 (47.6%)). Sex-specific differences were only seen for α-Klotho, in that term-born girls had higher α-Klotho levels than boys (*p* = 0.033). No further sex-specific differences were found (all remaining *p* > 0.05). When testing for between-group differences, significant differences between former preterm and term infants were detected for iFGF-23 (mean preterm: 43.2 pg/mL vs. term: 29.1 pg/mL; 95% confidence interval [CI] for the difference between means: 5.0 to 23.3 pg/mL; *p* = 0.003) and secretoneurin concentrations (median preterm: 93.8 pmol/L vs. term: 70.5 pmol/L; *p* = 0.046). Detailed information is provided in Fig. 1, Table 2 and Supplementary Fig. 1.

**Table 2:** Concentrations of hypoxia-inducible factor-1α (HIF-1α) and novel cardiovascular biomarkers at study visit in former term and very preterm infants.

Characteristic	Term (*n* = 21)	Preterm (*n* = 26)	*p*-value
HIF-1α [pg/mL]	< LOD; 3706.0	< LOD; 4360.5	0.179
iFGF-23 [pg/mL]	29.1 ± 10.5	43.2 ± 19.9	0.003**
cFGF-23 [pmol/L]	1.47 (1.07; 1.75)	1.24 (0.88; 1.94)	0.839
α-Klotho [pg/mL]	2242.7 ± 1031.2	1976.6 ± 558.9	0.330
Secretoneurin [pmol/L]	70.5 (62.3; 94.2)	93.8 (72.8; 110.3)	0.046*

Data are presented as total range or median (quartile 1; quartile 3) for non-normally distributed variables, and mean ± standard deviation (SD) for variables following a normal distribution. Plasma measurements were available in 47 of 47 subjects for HIF-1α, iFGF-23, and cFGF-23, 35 of 47 subjects for α-Klotho (term: 10/21 (47.6%), preterm: 25/26 (96.2%)), and 36 of 47 subjects for secretoneurin (term: 10/21 (47.6%), preterm: 26/26 (100%)). Data for HIF-1α analyses were censored as follows: limit of detection (LOD) = 81.92 pg/mL; census: LOD/√(2). **p < 0.01; *p < 0.05.

*HIF-1α* hypoxia-inducible factor-1α, *iFGF-23* intact fibroblast growth factor-23, *cFGF-23* c-terminal fibroblast growth factor-23.

### Echocardiographic measurements and aortic elastic properties in very preterm- and term-born children

Echocardiographic measurements were available in 39 of 47 subjects (preterm: 25/26 (84.6%), term: 14/21 (66.7%)), while measurements for aortic elastic properties were available in 42 of 47 subjects (preterm: 22/26 (84.6%), term: 20/21 (95.2%)). Measurements did not differ between male and female subjects (all *p* > 0.05). Significant differences between former preterm and term infants were detected for the distensibility of the descending aorta (ADD; mean preterm: 75.4 kPa^−1^ ×10^−3^ vs. term: 91.7 kPa^−1^ × 10^−3^; 95% CI for the difference between means: −28.7 to −4.0 kPa^−1^ ×10^−3^; *p* = 0.011). All analyses are listed in Table 3.

**Table 3:** Echocardiographic measurements and aortic elastic properties at study visit in former term and very preterm infants.

Measurements	Term (*n* = 21)	Preterm (*n* = 26)	*p*-value
EF [%]	67 (61; 69)	66 (62; 68)	0.747
FS [%]	35 ± 4	35 ± 3	0.881
LVM (Devereux) [g]	47.4 ± 10.1	44.0 ± 8.3	0.265
AAD [kPa^−1^ × 10^−3^]	67.1 ± 19.2	66.9 ± 17.4	0.973
AAS	3.4 (2.7; 4.4)	3.3 (3.0; 4.2)	0.850
ADD [kPa^−1^ × 10^−3^]	91.7 ± 22.3	75.4 ± 17.3	0.011*
ADS	2.7 (2.0; 3.2)	2.9 (2.4; 3.7)	0.065

Data are presented as median (quartile 1; quartile 3) for non-normally distributed variables, and mean ± standard deviation (SD) for variables following a normal distribution. Echocardiographic measurements were available in 39 of 47 subjects (term: 14/21 (66.7%), preterm: 25/26 (84.6%)), while measurements for aortic elastic properties were available in 42 of 47 subjects (term: 20/21 (95.2%), preterm: 22/26 (84.6%)). **p < 0.01; *p < 0.05.

*EF* ejection fraction, *FS* fractional shortening, *LVM* left ventricular mass (calculated according to Devereux), *AAD* aorta ascendens distensibility, *AAS* aorta ascendens stiffness index, *ADD* aorta descendens distensibility, *ADS* aorta descendens stiffness index.

### Association of novel cardiovascular biomarkers and echocardiographic measurements

Bivariate correlation analyses were performed to test whether plasma iFGF-23 or secretoneurin concentrations correlated with echocardiographic measurements. All analyses are provided in Table 4. Of note, iFGF-23 concentrations inversely correlated with ADD (Kendall-Tau-b τb = −0.240, *p* = 0.026, *n* = 42). A linear regression analysis was then used to investigate whether iFGF-23 concentrations were significantly associated with ADD in the overall study population. In a simple linear regression model, a significant association between iFGF-23 concentrations and ADD was found (R^2^ = 0.099, F_(1,40)_ = 4.41, ß = −0.363, *p* = 0.042). Multiple linear regression was further used to test if iFGF-23 and maturity at birth as a dichotomous variable were significantly associated with ADD. The overall regression model was statistically significant (R^2^ = 0.183, F_(2,39)_ = 4.368, *p* = 0.019), however neither iFGF-23 (*p* = 0.225) nor maturity at birth (*p* = 0.053) reached statistical significance.

**Table 4:** Correlations between echocardiographic measurements and intact fibroblast growth factor-23 (iFGF-23) as well as secretoneurin.

	iFGF-23 [pg/mL]	*p*-value	Secretoneurin [pmol/L]	*p*-value
EF [%]	0.096	0.390	0.199	0.104
FS [%]	0.126	0.260	0.224	0.067
LVM (Devereux) [g]	−0.077	0.497	−0.046	0.709
AAD [kPa^−1^ × 10^−3^]	−0.090	0.404	−0.047	0.709
AAS	0.101	0.356	0.078	0.537
ADD [kPa^−1^ × 10^−3^]	−0.240	0.026*	−0.212	0.088
ADS	0.163	0.134	0.235	0.064

Associations between two variables were assessed by means of Kendall’s tau-b (τb) correlation coefficient. Significance level was set at *p* < 0.05.

*iFGF-23* intact fibroblast growth factor-23, *EF* ejection fraction, *FS* fractional shortening, *LVM* left ventricular mass (calculated according to Devereux), *AAD* aorta ascendens distensibility, *AAS* aorta ascendens stiffness index, *ADD* aorta descendens distensibility, *ADS* aorta descendens stiffness index.

## Discussion

Owing to advancements in perinatal care,^52,53^ the majority of preterm infants nowadays reach adulthood.^5,6^ In view of the abovementioned facts pertaining to preterm birth rates and cardiovascular risk factors, increasing long-term morbidity in former preterm-born persons is on the cusp of becoming a public health challenge. Therefore, the elevated cardiovascular risk in this vulnerable population could further reinforce the significance cardiovascular disease is having on global morbidity and mortality.^54–56^ To the best of our knowledge, our study poses the first attempt to examine novel cardiovascular biomarkers and their association with incipient end-organ impairment utilizing non-invasive echocardiographic assessments in a population of very preterm-born children. To ensure representativeness of our sample, we compared our study cohorts to reference populations of former very preterm and term infants born within the same time frame. In the process of this analysis, no differences regarding distribution of sex, birth weight and gestational age were found (Supplementary Materials, Supplementary Tables 1 and 2). When comparing preterm and term infants within the study cohort, no differences in perinatal characteristics (except for the obvious difference in gestational age at birth and birth weight), characteristics at study visit, and routine laboratory parameters were found (see Table 1). Of relevance, both study groups had no comorbidities known to affect plasma concentrations of the biomarkers under investigation (i.e. normal kidney function, no signs of inflammation, anemia/increased erythropoiesis or iron deficiency).^33,36,57^

We found significantly higher concentrations of iFGF-23 and secretoneurin in former very preterm infants in comparison to term-born controls, while no differences in HIF-1α, cFGF-23 and α-Klotho were detectable. The increased levels of iFGF-23 were accompanied by lower plasma phosphate concentrations in the preterm group, but this finding did not reach statistical significance. Interestingly, the observed excess in iFGF-23 was not accompanied by α-Klotho deficiency, which points to an intact expression of Klotho and a potential compensatory effect.^33^ The observed higher concentrations of secretoneurin in preterm-born children in comparison to term-born controls reveal dissimilarities with our previous findings of lower secretoneurin concentrations in very preterm infants in umbilical cord blood and blood drawn at 48 h of life.^58^ But very preterm infants frequently experience hypoxemic episodes in the clinical course, and elevated secretoneurin concentrations later in life may be indicative of repeated insults throughout their stay in Neonatal Intensive Care.^58,59^

In echocardiographic assessments, we did not detect differences in estimates of ventricular systolic function or left ventricular mass, but did find significantly reduced aorta descendens distensibility (ADD) in former preterm infants. Further analyses revealed a significant inverse correlation of ADD with iFGF-23, which is in accordance with animal studies reporting reduced aortic relaxation in mice with elevated FGF-23 levels.^34^ The significant effect of iFGF-23 on ADD in the simple linear regression model was lost after controlling for preterm-born status in the multivariable regression. This could be the result of multicollinearity (of iFGF-23 and gestational age at birth) and/or further interpreted using the concept of mediation (with iFGF-23 as a possible mediator for the effect of gestational age on ADD). Additional larger studies (ideally with serial measurements) are required to disentangle direct and indirect effects as well as explore alternative mediators and confounders.

The limited sample size is the main limitation of our pilot study. This was partly accounted for by comparison to a reference population to ensure representativeness.

In essence, we offer first insights into the novel cardiovascular biomarkers FGF-23, α-Klotho and secretoneurin in a population of preterm-born children at kindergarten age. Our findings warrant replication in larger, more diverse study populations encompassing a broad range of gestational ages at birth and co-morbidities in the neonatal period, which may affect both biomarker concentrations and cardiovascular outcomes later in life.

In light of the increasing number of survivors of preterm birth and associated cardiovascular health challenges, early identification and initiation of tailored management in at-risk individuals is essential. Both iFGF-23 and secretoneurin hold promise as valuable cardiovascular biomarkers potentially allowing for risk stratification and timely implementation of preventive measures in those facing the most pronounced risk.

## Supplementary information

Supplementary Materials

## Data Availability

The datasets generated during and/or analyzed during the current study are available from the corresponding author on reasonable request.
