# Supplementary material for: Young hearts, early risks: novel cardiovascular biomarkers in former very preterm infants at kindergarten age
Source: Pediatr Res. 2024 Apr 24;96(4):999–1005. doi: 10.1038/s41390-024-03210-7 (PMC11502516; doi:10.1038/s41390-024-03210-7)

Supplementary Table 1: Comparison between the very preterm study cohort and a very preterm-born reference population

Characteristic	Study cohort (n = 26)	Reference population (n = 162)	p-value
Sex, male/female, N (%)	12 (46.2) / 14 (53.8)	80 (49.4) / 82 (50.6)	0.834
Gestational age [weeks]	29.5 (26.0; 31.4)	29.9 (28.4; 30.9)	0.715
Birth weight [g]	1204 ± 413	1254 ± 367	0.570

In order to ensure representativeness of the study sample, the very preterm study cohort was compared with a very preterm-born reference population (Innsbruck routine preterm follow-up database, birth dates 01/01/2007 – 07/31/2009). Categorical data are presented as counts (N) and percentages; continuous data are presented as median (quartile 1; quartile 3) for non-normally distributed variables or mean ± standard deviation (SD) for variables following a normal distribution.

Supplementary Table 2: Comparison between the term study cohort and a term-born reference population

Characteristic	Study cohort (n = 21)	Reference population (n = 9862)	p-value
Sex, male/female, N (%)	11 (52.4) / 10 (47.6)	4878 (49.5) / 4980 (50.5)	0.830
Birth weight [g]	3310 (3080; 3688)	3330 (3020; 3600)	0.622

In order to ensure representativeness of the study sample, the term study cohort was compared with a term-born reference population (SIDS database Tyrol, birth dates 01/01/2007 – 07/31/2009). Categorical data are presented as counts (N) and percentages; continuous data are presented as median (quartile 1; quartile 3) for non-normally distributed variables. The proportions of missing data in the reference population were as follows: 0.04% for sex, and 0.5% for birth weight.

Supplementary Figure 1: Plasma concentrations of intact fibroblast growth factor-23 (iFGF-23) (a), c-terminal FGF-23 (cFGF-23) (b), α-Klotho (c), and secretoneurin (d) in former extremely preterm infants (EPI) and very preterm infants (VPI) at kindergarten age. Each circle represents an individual measurement. Measurements in former EPI (<28 completed weeks of gestation at birth) are depicted as light-gray circles, measurements in former VPI (28 to 31+6 weeks of gestation at birth) as dark-gray circles. Biomarker concentrations are plotted on the y-axis in pg/mL and pmol/L, respectively. Center lines represent medians, whiskers mark 1st and 3rd quartiles. No significant differences in plasma biomarker concentrations at kindergarten age were detected between former EPI and former VPI (Mann-Whitney U Test, all $p > 0.05$). ns, not significant.

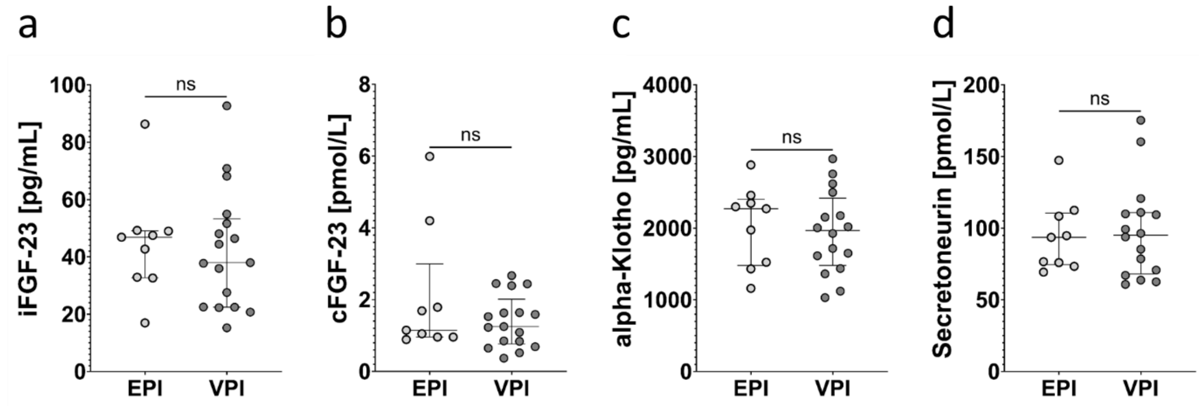

Supplement: Supplementary file 1 — Supplementary Materials [file 41390_2024_3210_MOESM1_ESM.pdf]
